# Systematic Review of Long Term Sinonasal Outcomes in CRSwNP after Endoscopic Sinus Surgery: A call for Unified and Standardized Criteria and Terms

**DOI:** 10.1007/s11882-024-01154-w

**Published:** 2024-06-24

**Authors:** Camilo Rodriguez-Van Strahlen, Claudio Arancibia, Christian Calvo-Henriquez, Joaquim Mullol, Isam Alobid

**Affiliations:** 1 Rhinology and Skull Base Unit, Department of Oto rhinolaryngology, Hospital Clinic Barcelona, Barcelona, Spain; 2 Service of Otolaryngology, Hospital Complex of Santiago de Compostela, Santiago de Compostela, Spain; 3 Institut d’Investigacions Biomèdiques August Pi i Sunyer (IDIBAPS), Barcelona, Spain; 4 Centro de Investigación Biomédica en Red de Enfermedades Respiratorias (CIBERES), Barcelona, Spain; 5 Unidad Alergo Rino, Centro Médico Teknon, Barcelona, Spain; 6 Universitat de Barcelona, Barcelona, Spain

**Keywords:** Long term follow up, Endoscopic sinus surgery, CRSwNP, Nasal polyp, Sinonasal outcome, Chronic rhinosinusitis, Review and meta-analysis

## Abstract

**Purpose of Review:**

To present current evidence in long-term (> 5 years) results after endoscopic sinus surgery (ESS) focusing on Patients Reported Outcome Measures (PROMs) and other sinonasal outcomes while assessing the role of ESS in the treatment of CRSwNP, and identifying outcomes which affect the results of ESS and defining recommendations for future studies.

**Recent Findings:**

Long-term results of ESS in CRSwNP can be branched in PROMs and other objective measurements. Despite the heterogeneity of reported outcomes make it difficult to perform comparisons and meta-analysis, ESS improves PROMs, including symptoms, QOL and olfaction. Objectives outcomes such as NPS, LMS, type of surgery, or recurrence and revision surgery don’t have a clear role in long-term results. Clustering patients suggest asthma, N-ERD, allergy, eosinophil count and IL-5 could have a role in predicting recurrence and severe disease.

**Summary:**

Long-term studies of CRSwNP treated with ESS are scarce. There is a significant need to standardize the report of results. The use of tools as SNOT-22, NPS, validated smell tests, defined criteria for disease recurrence and control and ESS extension in a unified systematic way could allow better comparisons between treatments in the new era of biologics.

## Introduction

Chronic rhinosinusitis with nasal polyps (CRSwNP) is an inflammatory disease affecting 0,5 − 4% [1, 2] of general population exerting significant socio-economic impact and profoundly influencing patients’ quality of life (QoL) [1, 3, 4]. CRSwNP is often linked to a type 2 endotype [1]. Type 2 inflammation is associated to a greater disease burden [3, 4] and is defined by tissue eosinophilia (≥ 10 eosinophils/high field power, blood eosinophilia (≥ 150 cells/µL and high level of serum total IgE (≥ 100 KU/L) and additional increased expression of T2 cytokines such as IL-4, IL-5, IL-13 [1, 5]. Comorbidities have a role in prognosis; asthma and non-steroidal exacerbated respiratory disease (N-ERD) condition worse outcome [6–10].

Currently, various treatment modalities exist for CRSwNP. Initially, these involve medical treatments, primarily centered on intranasal steroids and sinonasal rinsing. When medical treatment is ineffective or insufficient, therapy often turns to endoscopic sinonasal surgery (ESS) [1], which encompasses a diverse array of surgical techniques, improving symptoms and QoL [11–15]. In recent years, a novel treatment avenue has emerged in the form of monoclonal antibodies (mab). However, an ongoing debate persists regarding the specific role of surgery in the era of monoclonal antibodies. Resolving this issue hinges on addressing two crucial points.

Firstly, there is a need for a precise definition of surgical procedures. Notably, it is imperative to recognize that not ESS are identical; in fact, there is not current consensus on the optimal surgical strategy for CRSwNP [16]. Secondly, acquiring long-term data is essential as CRSwNP is an inflammatory chronic condition, and short-term outcomes may inadequately capture treatment efficacy in this disease. However, most reported results focus on short or median-term follow-ups [17].

This study aims to conduct a systematic review of the literature, providing a comprehensive overview of current evidence concerning sinonasal outcomes, particularly focusing on Patient-Reported Outcome Measures (PROMs), in long-term follow-ups (5 years or more) post-ESS. Additionally, it seeks to elucidate critical parameters for CRSwNP follow-ups and undertake a meta-analysis to delineate recommendations and shed light on the long-term outcomes of ESS.

### Search and Methodology

The review was performed according to PRISMA 2020 guidelines [18]. The search was conducted in October 2023 in the electronic databases Medline/Pubmed, Cochrane Library and Scopus.

#### Search Strategy, Inclusion, and Exclusion Criteria

The parameters considered for the systematic review were based on population, intervention, comparison, and outcome (PICOTS) framework [19] as follows:

- Participants: CRSwNP patients,
- Intervention: undergoing ESS,
- Comparison: before ESS and long-term follow-up results (≥ 5 years) and.
- Outcomes: sinonasal outcomes, focused on PROMs,
- Time and setting: without limitation.

The inclusion criteria were the following: (1) Type of study: clinical trials, case series, prospective and retrospective cohort studies published in peer-reviewed journals. (2) The search had no restriction regarding date, all publications before October 2023 were included. The exclusion criteria consisted of (1) Mean or median follow up less than 5 years or 60 months. (2) No PROMs or sinonasal outcomes reported. (3) Mean or median follow up period not reported or unable to estimate specifically for CRSwNP patients. (4) studies with subjects < 18 years old. (5) Studies that performed or mix procedures different to ESS, such as headlamp nasal polypectomy, in-office, or balloon sinuplasty. (6) study type: case reports, review articles, letters to the editor, theses, and meeting communications.

The search strategy used was the following: ((((((((((((CRSwNP[Title/Abstract]) OR (chronic rhinosinusitis nasal polyps[Title/Abstract])) OR (chronic nasal polyps[Title/Abstract])) OR (chronic nasal polyposis[Title/Abstract])) OR (chronic rhinosinusitis nasal polyposis[Title/Abstract])) OR (chronic rhinosinusitis sinonasal polyposis[Title/Abstract])) OR (chronic sinus polyposis[Title/Abstract])) OR (chronic rhinosinusitis with nasal polyps[Title/Abstract])) OR (chronic rhinosinusitis[Title/Abstract] AND nasal polyps[Title/Abstract])) OR (chronic rhinosinusitis with nasal polyposis[Title/Abstract])) OR (chronic rhinosinusitis[Title/Abstract] AND nasal polyposis[Title/Abstract])) OR (chronic rhinosinusitis associated sinonasal polyposis[Title/Abstract])) AND (((((ESS[Title/Abstract]) OR (Endoscopic sinus surgery[Title/Abstract])) OR (FESS[Title/Abstract])) OR (Functional endoscopic sinus surgery[Title/Abstract])) OR (Reboot surgery[Title/Abstract])).

**Fig. 1:**
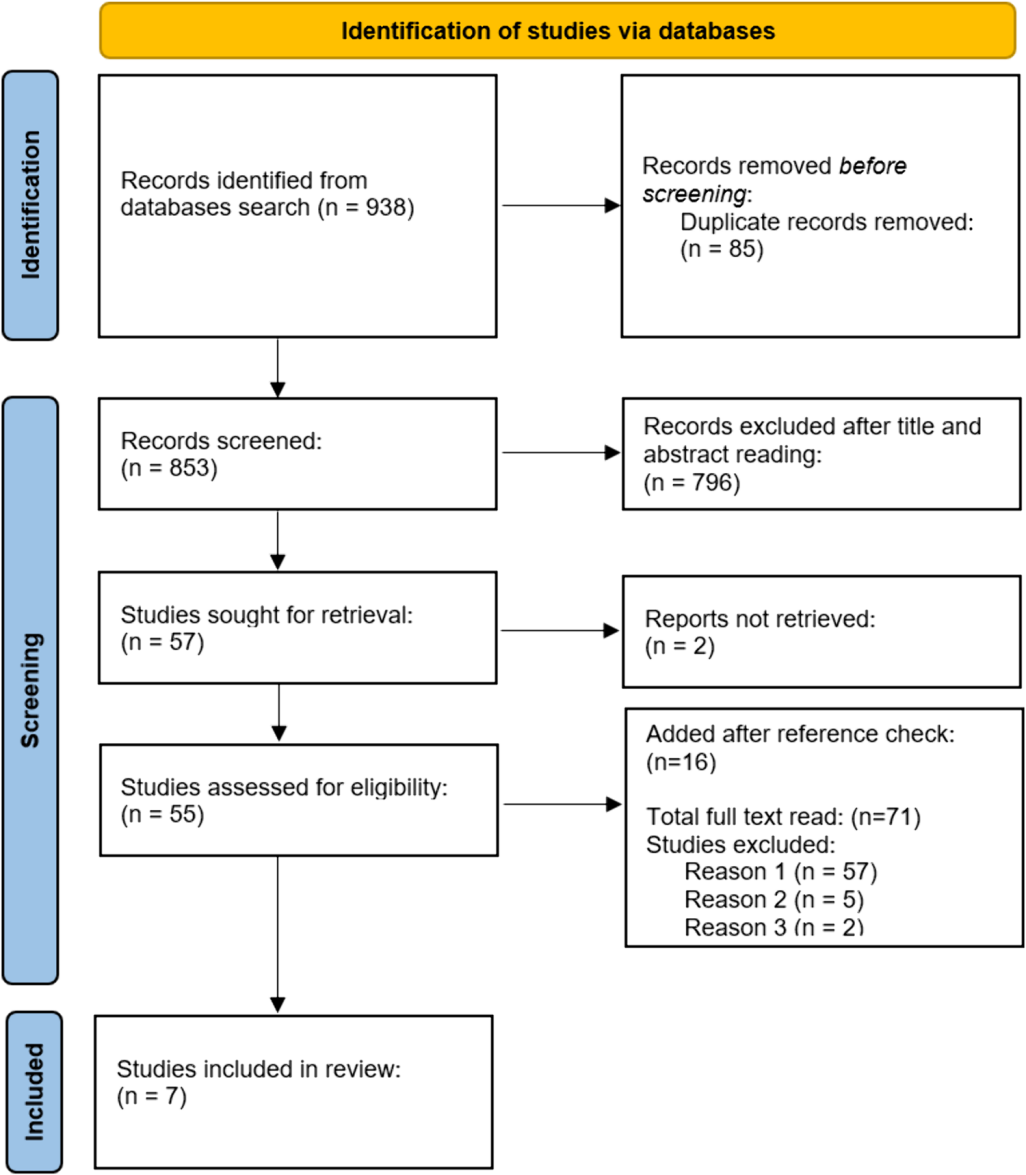
PRISMA flow diagram for the selection of the original articles for the review

PRISMA flowchart summarizes the search process (Fig. 1). Initial search retrieved 938 articles. Titles and abstracts were screened in duplicate (CRV and CA) after the initial screening the articles were selected for full text read.

Full read papers (*n* = 57) were mainly excluded for the following reasons: [1] Minimum mean/median follow-up. Either not reported, shorter than 5 years or 60 months, or incomplete information regarding follow-up time (*n* = 41) [2]. CRSwNP group related information. After detailed reading, the article didn’t specify PROMs or sinonasal outcomes specifically for CRSwNP group of patients, or it wasn’t possible to estimate it with the available information (*n* = 5) [3]. Adequate surgical techniques. This revision is focused on ESS only. Headlamp surgery or polypectomies, in-office procedures and sinus ballon techniques were excluded from the analysis (*n* = 2).

After full text read the reviewers manually checked the references list of all articles to include any suitable article under looked during the search. The outcomes were extracted from each study and presented as “previous to ESS” (T0) and “after ESS” (T1) considering as T1 the results from the longest follow-up reported.

Statistical analysis was performed by the method assistant (CCH). All statistical data were analyzed using STATA for Macintosh v. 15.1 (StataCorp®). Significance was considered at a P-value < 0.05.

The Cochrane tool to estimate the mean and standard deviation from median and interquartile range was used from the Cochrane handbook for systematic reviews [20].

We used the rBiostatistic web tool to conduct the meta-analysis (https://www.rbiostatistics.com/one_group_means*).* Heterogeneity was assessed using the Q-test and I2 test. A fixed-effects model was used when the I2 heterogeneity value was < 50% and *p* ≥ 0.05, and a random-effects model when I2 was ≥ 50% and *p* < 0.05. Finally, publication bias was assessed using a funnel plot and Egger regression.

## Results

A total of 7 articles were included adding a total of 1076 patients with long-term follow-up after endoscopic sinus surgery. The mean sample size was 153.7 patients, but with a high dispersion as the smallest included 38 participants [21] and the largest 540 [22].

The mean weighted mean age was 47.6, being the lowest 45.4 [23] and the highest 49.4 [24]. It is noteworthy that only 4 studies could be included in this analysis, as Calus et al [21] only provided medians, while Zhang et al. and Mendelsohn et al [16, 22] only data from individual groups.

**Fig. 2:**
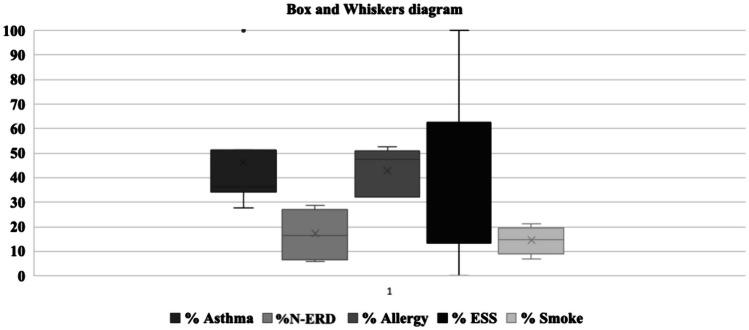
Box and whiskers diagram. Percentage of patients presenting potential confounding factors in studies included. Asthma; N-ERD (non-steroidal exacerbated respiratory disease); allergy; previous ESS (endoscopic sinus surgery); Smoking

Regarding categorical variables (present/absent) there was an overall similarity between studies, Fig. 2 displays important characteristics from cohorts. Asthma was similar between studies (45.8%), but there was an outlier with 100% prevalence [16]. The prevalence of previous ESS was the most disperse variable, with results ranging from 0% [25] to 100% [16]. Demographic data of cohorts is detailed in Table 1.

**Table 1:** Demographic results of long-term cohort subjects with CRSwNP with complete ESS follow-up

**Author and year**	**Complete follow-up**	**Male** ***N*** **(%)**	**Female** ***N*** **(%)**	**Mean Age, years.** **Mean or median (SD or IQR)**	**Asthma** ***N*** **(%)**	***N*-ERD** ***N*** **(%)**	**Atopy & Method** ***N*** **(%)**	**Previous ESS** ***N*** **(%)**	**Smoking** ***N*** **(%)**
**Arancibia C** **2022**	39	27 (69.2)	12 (30.8)	45.4 (10.8)	19 (51.3)	10 [11]	NR	7 (17.9)	NR
**Simmonds JC** **2022**	184.2^a^	85.5^a^ (46.4)	98.7^a^ (53.6)	46.9 (Range: 18–80)	(34.1)	(6.4)	NR (49.5)	(47.4)	(6.8)
**Riva G** **2022**	62	42 (68.9)	19 (31.1)	47.1 (11.2)	22 (36.1)	10 (16.4)	29 NR (47.5)	0	9 (14.8)
**Vlaminck** **2022**	133	83 (62.4)	50 (37.6)	49.4 (12.2)	37 (27.8)	8 [6]	43 SPT (32.3)	30 [6, 22]	NR
**Zhang L** **2020**	81	48 (50.3)	33 (49.7)	FESS 44.6 (11.2)	81 (100)	24 (28.6)	26 ImmunoCap (32.1)	81 (100)	12 (14.8)
				RESS 47.30 (11.0)					
				RESS + D3 41.4 (12.8)					
**Calus L** **2019**	38	25 (65.8)	13 (34.3)	Primary ESS 44 [30–53]	15 (39.5)	10 (26.3)	20 SPT (52.6)	19 [49]	NR
				Revision ESS 47 [36–52]					
**Mendelsohn** **2011**	540	303 (59.4)	237 (40.6)	CRSwNP 54	187 (35.4)	61 (11.3)	NR Excluded from analysis	NR	114 (21.1)
				CRSwNP/A 56					
				CRSwNP/AA 55					

*CRSwNP *Chronic Rhinosinusitis with Nasal Polyps*, CRSwNP/A *CRSwNP and Asthma*, CRSwNP/AA *CRSwNP and Asthma and N-ERD*, ESS *Endoscopic Sinus Surgery*, FESS *Functional Endoscopic Sinus Surgery*, ImmunoCap (Immuno-Cap Phadiatop (Pharmacia, Uppsala, Sweden) (cutoff ≥ 0.35 kU/mL)), IQR *Interquartile Range*, N-ERD *Non-steroidal anti-inflammatory exacerbated respiratory disease*, NR *Not reported*, RESS *Radical Endoscopic Sinus Surgery*, RESS + D3 *Extended Endoscopic Sinus Surgery*, SD *Standard Deviation*, SPT *Skin Prick Test performed to define allergy status

^a^ *(Data estimated from article data, no statistical significance difference between CRSwNP and CRSsNP group is reported in preoperative evaluation)*

### Long-Term Sinonasal Outcomes of CRSwNP Patients after ESS

Table 2 shows data extracted from the seven studies fulfilling the search criteria. The heterogenicity of variables measured is detailed in *“variable”* column. Next, results are presented in relation to an array of variables.

**Table 2:** Long-term sinonasal outcomes of CRSwNP patients after ESS: PROMs and objective measures

Author and year	Follow up time. (mean or median and *n*)	Sinonasal Outcomes Assessed		
		Variable	T0	T1
**Arancibia C** **2022**	5 years (*n* = 39)	T5SS NPS BAST-24 • Detection • Memory • Identification	13 (IQR 10–14) 8 (IQR 6–8) ------ • 0 (IQR 0–5) • 0 (IQR 0–5) • 0 (IQR 0–5)	6.5 (IQR 4–9)^a^ 1 (IQR 0–3.3)^a^ ------ • 55% (IQR 0-100)^a^ • 37,5% (IQR 0-100)^a^ • 15% (IQR 0–50)^a^
	12 years (Med: 12y (IQR: 11–14)) (*n* = 39)	T5SS NPS BAST-24 • Detection • Memory • Identification LMS Symptom recurrence NP/Symptom recurrence Revision surgery	13 (IQR 10–14) 8 (IQR 6–8) ------ • 0% (IQR 0–5) • 0% (IQR 0–5) • 0% (IQR 0–5) 20 (IQR 15–22) NA NA NA	6 (IQR 3–9)^a^ 2 (IQR 1–3)^a^ ------ • 65% (IQR 0-100)^a^ • 15% (IQR 0-46.2) • 30% (IQR 0–55)^a^ 12 (IQR 9.2–15)^a^ 32 (82%) 82.1% 7 (21.9%)
**Simmonds JC** **2022**	5 years (*n* = 184.2)^b^	SNOT22^b^	48.5^b^ (SE: 0.7)	25.8^b^ (SE: 1.1)^a^
**Riva G** **2022**	5 years (*n* = 62)	Recurrence rate	NA	30.3%
	10 years (Me: 126.49 ± 60.9mo (range 39–267 months)). (*n* = 62)	Recurrence rate Time to recurrence	NA NA	66.06% 106mo (IQR 99)
**Vlaminck S** **2021**	13.5 years (Me: 13.5 y (SD: 1.9)) (*n* = 133)	SNOT-22 NP/Symptom recurrence Revision surgery Time to recurrence	NR NA NA NA	24,9 (SD ± 18.2) 82 (62%) 34 (26%) 4.6 y (SD ± 4.9)
**Zhang L** **2020**	5 years (Mean and SD not specified) FESS (*n* = 27) RESS (*n* = 27) RESS + Draf3 (*n* = 27)	NP score (0–3, max 6) SNOT-22 • FESS • RESS • RESS + Draf3 Nasal Symptoms (VAS) -Congestion • FESS • RESS • RESS + Draf3 -Rhinorrhea • FESS • RESS • RESS + Draf3 -Loss of smell • FESS • RESS • RESS + Draf3 -Headache • FESS • RESS • RESS + Draf3 Lund-Kennedy • FESS • RESS • RESS + Draf3 LMS • FESS • RESS • RESS + Draf3 Recurrence Revision Surgery Time to recurrence • FESS • RESS • RESS + Draf3	5;5;5 ------ • 64.0 (IQR 61.0–67.0) • 65.0 (IQR 57.0–68.0) • 64.0 (IQR 54.0–75.0) ------ ------ • 8 (IQR 7.8–8.4) • 8 (IQR 7.5–8.4) • 8 (IQR 7.7–8.5) ------ • 4 (IQR 4.0–5.0) • 5 (IQR 4.0–5.0) • 5 (IQR 4.0–6.0) ------ • 10 (IQR 9.0–10.0) • 10 (IQR 9.0–10.0) • 9 (IQR 9.0–10.0) ------ • 3 (3.0–3.0) • 3 (3.0–3.0) • 3 (2.0–4.0) ----- • NR • NR • NR ------ • 20 (IQR 18.0–23.0) • 20 (IQR 17.0–24.0) • 22 (IQR 18.0–24.0) NA NA ------ NA NA NA	NR ------ • 42^b^ • 44^b^ • 40^b^ ------ ------ • 5^b^ • 5^b^ • 5^b^ ------ • 3.5^b^ • 3^b^ • 4^b^ ------ • 7^b^ • 7^b^ • 7^b^ ------ • 2^b^ • 2^b^ • 2^b^ ------ • 14^b^ • 14^b^ • 14^b^ ------ • NR • NR • NR 95,9% 27% ------ • 9 m [6–9] • 12 m [9–18] • 12 m [6–19]
**Calus L** **2019**	12 years (Mean and SD not specified) (*n* = 38)	Total Symptom Score -Smell disturbance^a^ • No • Mild • Moderate • Severe -Nasal obstruction^a^ • No • Mild • Moderate • Severe -Rhinorrhea • No • Mild • Moderate • Severe -Headache • No • Mild • Moderate • Severe -Sneezing • No • Mild • Moderate • Severe -Eye symptoms • No • Mild • Moderate • Severe NPS • 0 • 1–2 • 3–4 • 5–6 NP only Recurrence Revision surgery	8 (IQR 7–11)^b^ • 2.7% • 5.4% • 18.9% • 73% ------ • 0% • 10.8% • 29.7% • 59.5% ------ • 27% • 21.6% • 24.3% • 27% ------ • 37.8% • 21.6% • 21.6% • 18.9% ------ • 37.8% • 27% • 29.7% • 5.4% ------ • 78.4% • 18.9% • 2.7% • 0% 4 (IQR 3–6) 0% • 15.8% • 42.1% • 42.1% NA NA	Not reported • 28.9% • 12.8% • 10.5% • 34.2% ------ • 39.5% • 34.2% • 21.0% • 5.3% ------ • 42.1% • 39.5% • 15.8% • 2.6% ------ • 60.5% • 26.3% • 10.5% • 2.6% ------ • 50% • 42.1% • 5.3% • 2.6% ------ • 65.8% • 26.3% • 2.6% • 5.3% Not reported 40% • 40% • 14.3% • 5.7% 78.9% 36.8%
**Mendelsohn** **2011**	5 years (Mean and SD not specified)	Polyp recurrence •CRSwNP •CRSwNP and asthma •N-ERD	•NA •NA •NA	•16% •45% •90%

*AESS *Additional Endoscopic Sinus Surgery, *BAST-24 *Barcelona Smell Test 24, *CRSwNP *Chronic Rhinosinusitis with Nasal Polyps, *eCRSwNP *Eosinophilic CRSwNP, Eos Eosinophil, *ERM *Eosinophil Rich Mucus, *FESS *Functional Endoscopic Sinus Surgery, *IQR *Interquartile range, *LMS *Lund Mackay system, *LMS *Lund-Mackay score, *Me *Mean, *Med *Median, *NA *Not Applicable, *neCRSwNP *non-eosinophilic CRSwNP, *NERD *Non-steroidal anti-inflammatory exacerbated respiratory disease, *NP *Nasal polyp, *NPS *Nasal polyp score, *NR *Not reported, *PROM *Patient reported outcome measure, *RESS *Radical endoscopic sinus surgery, *SD *Standard deviation, *SE *Standard error, *SNOT-22 *Sinonasal Outcome Test 22, *SP *Simple polypectomy, *T5SS *Total 5 symptoms score, *UD *Uncontrolled Disease according to EPOS2022

^a^* (Statistical significance reported in the result)*

^b^ *(Estimated data extracted from numbers or graphs)*

#### Symptoms and Quality of Life Questionnaires

Four from the seven selected studies [16, 23, 24, 26] assessed quality of life questionnaires. Three [16, 24, 26]of them used the SNOT-22, while Arancibia et al [23] used T5SS. The three SNOT-22 studies could not be pooled in a meta-analysis, as only Simmonds et al. provided their whole data (mean and standard deviation before and after ESS). There was significant dispersion between results, as postoperative SNOT-22 vary from around 25 [24, 26] to more than 40 [16]. Table 2 compiles symptoms assessment done by five authors [16, 21, 23, 24, 26].

#### Olfaction

Only one study instrumentally assessed olfaction [23] using the BAST-24 validated smell test, while two studies [16, 21] report it as a self-reported symptom after long-term follow-up (5–12 years). All studies assessing olfaction [16, 21, 23]report improvement in long-term measurements (BAST-24,VAS scale or other) after ESS, one finds significant improvement after ESS at 5y (*p* < 0.006) and 12y (*p* < 0.031) follow up [23]. However, given the heterogeneity, no meta-analysis can be performed and no data can be compared. Preoperative instrumental test (Arancibia et al.) seems to correlate to self-reported symptoms [16, 21, 23].

#### Recurrence of Disease

Six studies report recurrence [16, 21–25]. However, the definition of recurrence was remarkably different between studies (Table 3), which prevents us from performing comparisons or meta-analysis. The methods followed to define recurrence are summarized in Table 3. Only Arancibia et al. reported their recurrence according to symptoms. Three studies [21–23] report nasal polyp recurrence but following different definitions. Vlaminck et al. [24] study mixes in the same category nasal polyps and symptom recurrence. While other 2 studies [16, 25] do not report how recurrence was defined. This lack of homogeneity is reflected in recurrence reports varying from 16% [22] to 95.9% [16].

**Table 3:** Characteristics of studies included (design, groups, interventions, and definition of recurrence)

**Study**	**Design of study**	**CRSwNP criteria**	**Type of CRSwNP**	**Described surgical technique**	**PostOp treatment**	**Definition of recurrence**
**Arancibia C** **2022**	Prospective cohort	EPOS 2005	Refractory moderate to severe CRSwNP	NPmy, MxAn, AntEth PostEth, SphAn, Draf I or IIa or IIb or III.	Nasal rinsing INC	Nasal endoscopy and symptoms assessment.
**Simmonds JC** **2022**	Prospective cohort	NR	Refractory CRS	ESS. Extend of surgery was determined by primary surgeon.	NR	NR
**Riva G** **2022**	Retrospective Cohort	EPOS 2020	Refractory CRSwNP	NPmy, MxAn, AntEth +/- other (not specified)	Nasal rinsing INC	Not specified
**Vlaminck 2021**	Prospective cohort	EPOS 2012	Moderate to severe CRSwNP	MxAn (wide), AntEth, PostEth, SphAn (wide), Draf I or IIa.	Nasal rinsing INC Ointment +/- ATB and SCS if inflammation persisted 4–6 weeks after surgery	Mucosa inflammation, NP, mucopurulent secretions. At least 3 symptoms: nasal obstruction, rhinorrhea, postnasal drip, facial pain/headache, smell disturbance, sleep disturbance/fatigue) for 1 month
**Zhang L** **2020**	Prospective cohort RCT	EPOS 2007	Refractory CRSwNP EPOS 2007	NPmy, MxAn, AntEth, PostEth, SphAn. + 1 of the following: • FESS (with Draf I) • RESS (SphAn (wide), Draf IIa, MT partial resection) • RESS + D3 (SphAn (wide), Draf III, MT partial resection)	Nasal rinsing INC ATB 10 days. SCS: 3 weeks methylprednisolone	Not specified clearly in the article.
**Calus L** **2019**	Prospective cohort	EPOS 2012	Moderate to severe CRSwNP	NPmy, MxAn, AntEth, PostEth, SphAn, Draf I +/- MT partial resection.	Nasal rinsing INC ATB or SCS at variable doses.	NPS > 0 and EPOS 2012 control test.
**Mendelsohn** **2011**	Retrospective cohort	AAO definition of CRSwNP of 1997	CRSwNP with failed full medical therapy	NPmy, MxAn, AntEth, PostEth, SphAn, Draf I	NR	NP after surgery.

*AAO *American Academy Of Otolaryngology - Head And Neck Surgery, *AntEth *Anterior Ethmoidectomy, *ATB *Antibiotic, *CRS *Chronic Rhinosinusitis, *CRSwNP *Chronic Rhinosinusitis with Nasal Polyps, *EPOS *European Position Paper on Poliposis, *ESS *Endoscopic Sinus Surgery, *INC *Intranasal Corticosteroids, *MT *Middle Turbinate, *MxAn *Maxillary Antrostomy, *NP *Nasal Polyp, *NPmy *Nasal polypectomy, *NPS *Nasal Polyp Score, *NR *Not reported, *PostEth *Posterior Ethmoidectomy, *RCT *Randomized controlled trial, *SCS *Systemic corticosteroids, *SphAn *Sphenoidotomy

#### Revision Surgery

Four studies [16, 21, 23, 24] report their revision surgery rate (gathering 291 patients). It ranged from 21.9% at 12 years [23] to 36.8% at 12 years [21]. As the definition of revision surgery is the same, data can be gathered. The combined proportion (weighted by their sample size) of revision surgery after long-term follow up is 27.14%.

#### Nasal Polyp Size

Only two authors report nasal polyp size [21, 23] at long-term after ESS. Both used the NPS. However, Calus et al [21] only report the pre-surgery data, but not the postoperative data, so their data cannot be combined in a meta-analysis.

## Discussion

To the best of our knowledge, this is the first systematic review assessing long-term sinonasal outcomes after ESS focusing exclusively on CRSwNP. However, conducting a meta-analysis was not feasible due to the heterogeneity of the published data.

This review contributes to evaluating ESS’s role in the treatment of CRSwNP, especially considering the introduction of new therapies as mab. ESS is a safe and effective procedure [1, 6, 11, 12] however, in the coming years, its specific role in the CRSwNP treatment algorithm needs to be determined. High-quality data is very important in order to compare treatment options, short- and long-term outcomes. For example, while extended surgery seems to yield better short-term results, its long-term efficacy is less conclusive [16, 27]. Further studies are needed to clarify this specific aspect.

This review highlights the extreme need for a collective effort to obtain new high-quality long-term data. Despite CRSwNP being a highly prevalent disease and ESS being widely performed, only seven studies could be included in this review. Among them, no statistical comparisons or common analyses could be performed due to the heterogeneity in assessing the different outcomes. This also highlights the lack and urgent need to define common methods for determining outcomes. This review has been focused on different outcomes that are summarized and discussed, main findings show that SNOT-22 emerges as an easy, adequate, and efficient tool to assess outcomes, regarding olfaction the use of validated tests is recommended to evaluate treatment results and QoL. NPS should be reported before and after any treatment during follow-up, Gevaert et al. [28] publication provides guidance in this matter. The concept of recurrence should be replaced by control of disease, using specific criteria and methodology to allow comparison of results. Revision surgery in long-term follow-up was 27%. Lastly patient clustering and cofounding factors can arise in the follow-up of CRSwNP. Factors as asthma and N-ERD play a role in disease prognosis. Others as atopy, extent of surgery, presence of previous surgery and IL-5 need to be clarified and are of interest in future research.

### Nasal Symptoms and QoL

Surprisingly, regardless of the outstanding importance of nasal symptoms and QoL for the otorhinolaryngologist, only 4 studies assessed these variables [16, 21, 23, 26]. Nasal symptoms are reported using numerous PROMs, making comparisons difficult. Among the different PROMs, SNOT-22 stands out as the most spread and validated. It is an outstanding tool to assess nasal symptoms and QoL associated to nasal conditions [29, 30]. Correct interpretation of SNOT-22 should be comprehensive, variables as gender and some comorbidities can influence its result [16, 26, 31].

Standardizing its use, as suggested by EPOS 2020 and the POLINA 2.0, can facilitate comparisons and treatment decisions [1, 32]. We strongly suggest performing SNOT-22 in our daily practice to have data for future long-term studies.

### Olfaction

When assessing independent symptoms, olfaction stands out as one of the most influencing symptoms on QoL. Therefore, it should be a primary outcome in any CRSwNP treatment. However, only three studies [16, 21, 23] (Arancibia, Calus, Zhang) reported olfaction data after long-term follow-up, while only one single study used a validated olfaction test (BAST-24 [33]). Their data is encouraging, as all the studies included report a positive and sustained effect on long-term olfaction [16, 21, 23–26]. We strongly support performing instrumental testing in the follow-up of CRSwNP patients.

### Nasal Polyp Score

Despite the main objective when treating CRSwNP is symptom control, regardless of the nasal polyp size, it is still a remarkable outcome variable. Nasal polyp size has been assessed through various scales, with the most recent being the system introduced by Meltzer in 2006 and a similar system employed in multiple clinical trials [34–43]. Efforts to establish a standardized reference for endoscopic CRSwNP scoring have been published, facilitating cross comparisons [28]. In this review, only two authors [21, 23] (Arancibia and Calus) reported the Nasal Polyp Score (NPS), but limited preoperative data precludes in-depth analysis. It’s worth noting a recent meta-analysis found no significant association between NP scoring systems and PROMs like SNOT-22, Total Nasal Symptom Score (TNSS), nasal congestion scores, and objective olfaction measurements (SIT-40) [44]. However, no long-term analysis could be performed.

### Recurrence After ESS: Definition of Controlled Disease

Recurrence after long-term follow-up has been described in six studies. However, yet, there is no common criteria of what should be defined as “recurrence”. In fact, in EPOS2020 [1], CRS control is defined as: “A disease state in which the patients do not have symptoms, or the symptoms do not adversely affect quality of life, if possible combined with a healthy or almost healthy mucosa and only need for local medication”. However, this definition, despite correct is too vague and does not allow for a clear definition of recurrence or disease control. This vagueness is reflected in this review, as all the authors used a different definition of recurrence.

In fact, the concept of “recurrence” may not be suitable for CRSwNP since the absence of nasal polyps after ESS doesn’t imply the cure of the underlying inflammatory condition. The terms “Controlled,” “Partly controlled,” and “Uncontrolled” could be more suitable to categorize patients [1, 32]. POLINA [32] defines control using VAS, SNOT-22, NPS, use of oral steroids and need for surgery. We strongly suggest future publications to use a clear methodology to define illness recurrence/control.

### Revision Surgery

Revision surgery was reported in 4 studies included in this systematic review [16, 21, 23, 24]. Long-term revision surgery rate with pooled data was 27% out of 291 patients with CRSwNP. Revision surgery rate can be easily defined, and this data can be effortlessly compared to any other long-term CRSwNP treatment database. There has been a previous meta-analysis assessing this very topic reporting revision rates between 14 to 24% [45]. However, revision surgery in that meta-analysis was not focused on long-term, while it mixed long- and short-term results.

### Patient Clustering and Confounding Factors

As with any other systematic review, the differences in the confounding factors may alter our ability to compare results from different studies. Here on, different potential confounding factors are discussed.

#### Type and Extent of Surgery

The first and most important confounding factor is the type of surgery. Variable definitions of surgical techniques in the literature emphasize the need for standardized terminology. This is highly relevant, as it has been shown that more extensive surgery is less prone to revision surgery [11]. The “radical surgery” concept [1] showing short- and mid-term benefits over FESS in reducing recurrence and improving symptoms and smell [16, 46–50]. There is an urgent need to define common terminology to define ESS. Nowadays there is the ACCESS project [51], to define whether a surgery was sufficient. Pending of publication is the LOEM classification [52], which aims at helping in accurate description of the surgery. We strongly suggest following studies to clearly define their surgeries to allow comparisons in the future.

#### Asthma

In long-term follow-up asthmatic patients exhibit poorer outcomes in CRSwNP, given by higher recurrence rates (symptomatic and endoscopic), increased need for medication, impaired smell, reduced QoL, increased disease burden, and elevated revision ESS rates [16, 21, 23–26, 45, 53–56]. While not all studies show statistical significance, Simmonds et al. notes a trend toward worse SNOT-22 scores [26]. Arancibia et al. highlighted significant worse results in loss of smell, symptoms, and a 100% recurrence rate at 12-year follow-up for the asthmatic group [23]. Vlaminck et al. reports significantly higher recurrence rates for asthmatics compared to non-asthmatics [24]. Smith et al. associated asthma with increased risk of 2nd and 3rd ESS (*p* < 0.001) [54]. Asthma may be considered a risk factor for recurrence and symptom control in CRSwNP patients treated with ESS.

#### Non-Steroidal Exacerbated Respiratory Disease (N-ERD)

It well known N-ERD patients exhibit elevated recurrence and revision surgery rates [55–58] as higher revision surgery in the frontal sinus [59]. Notably, Riva et al. identified a higher recurrence per year (*p* = 0.035), indicative of multiple recurrences [25]. Mendelsohn et al. detected earlier and increased recurrence (*p* < 0.001), higher secondary revision ESS (*p* < 0.001), and is a risk factor for nasal polyp recurrence (*p* < 0.01) and revision surgery (*p* < 0.01) [22]. Although further robust studies are essential for confirmation, N-ERD appears however as a long-term predictor of recurrence and disease burden.

#### Atopy (Allergen Sensitization)

Overall, the association between atopy and long-term outcomes after EES in CRSwNP is complex, with some studies suggesting a significant relationship. Skin Prick Test (SPT) or ImmunoCap do not allow to differentiate atopy (allergen sensitization) from allergic disease. In CRSwNP, interrogation for allergen related symptoms is difficult and was not specified by any of the studies. Simmonds et al. [26] links atopy better outcomes after surgery regarding SNOT-22. Vlaminck et al. [24] describes higher prevalence of atopy in recurrence group. Calus et al. [21] states allergen sensitization status as predictor for revision surgery (OR 6.1) with shorter time to ESS. Riva et al. [25] doesn’t find significant differences. Further research with comprehensive allergen testing, adequate clarification of allergic disease and larger sample sizes are needed to clarify these relationships and their impact on long-term surgical outcomes in CRSwNP.

#### Eosinophils

Blood eosinophil count (BEC), and other eosinophil derived measures (Eosinophil rich mucus, eosinophilic nasal cytology, Eosinophilic cationic protein) have been previously associated with extensive disease, revision surgery and recurrence [60, 49]. In long-term follow-up after ESS in CRSwNP, BEC has a moderate correlation to higher scores in PROM (T5SS) in the asthma subgroup at 12 years [23]. Recurrence rate was higher in patients with local eosinophilia and eosinophilic rich mucus(*p* < 0.001) [24]. In the long-term most authors did not assess nor analyze eosinophil derived outcomes. The role of eosinophils in predicting outcomes, particularly in asthmatic patients, appears noteworthy, but further research and a comprehensive analysis across various parameters are necessary for a more accurate understanding.

#### Interleukin 5

Concerning long-term outcomes following ESS in CRSwNP, Calus et al [21] revealed a significant association between high levels of IL-5 and patients with ESS history. Elevated IL-5 may be used as a modest predictor of revision ESS during the follow-up period (OR 1.004, 95% CI 1.001 to 1.008, *P* < 0.05). Although no significant association between IgE and revision ESS could be found. this finding shows a prognostic significance of IL-5 at long-term follow-up, offering insights into the potential use of mab as a preventive measure against the need for revision surgery in CRSwNP patients.

#### Previous ESS

Previous ESS predicts revision surgery and worse outcomes in CRSwNP, shown by higher recurrence in those with prior ESS (*p* < 0.004) [23, 24], and a trend towards worse SNOT-22 outcomes [26]. Senior et al. [17] noted a trend for revision surgery in patients with prior procedures, contrasting with Hopkins et al. [11], stating patients with nasal polypectomy and additional ESS are less likely to undergo revision surgery (adjusted OR 0.66; *p* = 0.04). Zhang et al.’s RCT [16] with high-risk factor for recurrence showed RESS (with or without Draf3) having better short-term outcomes (1 year), but at 5 years, RESS and RESS + D3 appear similar. Arancibia performed frontal sinusotomy in 94.9%, 3 patients with extended ESS [23], and Simmonds et al. [26] reported 62.3% frontal sinusotomy. CRSwNP patients have a higher chance of frontal sinusotomy at the original surgery (*p* < 0.001) [54]. Evidence suggests that previous surgery in CRSwNP may serve as a predictor for future revision surgery and worse PROMs.

### Limitations

As with any systematic review, some papers may have been missed. The main limitation of our review, and extensively discussed in the manuscript, is the heterogeneity of the methods employed to express the different outcomes, which have limited us to compare results. Despite considering it as limitation, we believe that this should be consider a main finding of our review, highlighting the urgent need of standardizing the way of presenting CRSwNP outcomes and results to enable comparisons between treatments and surgical techniques.

The third limitation of this review is the small number of included papers. Again, it is certainly a limitation for obtaining solid information, but it is itself a main finding of our review. This shortness of long-term follow-up studies highlights the urgent need for this type of studies. Lastly, there is inaccuracy discerning atopy (allergen sensitization) from allergic disease in CRSwNP patients. Long-term studies don’t provide enough information to categorize patients as allergic (symptoms are not directly related and developed after exposure to allergen); due to this limitation, the role of allergic disease cannot be assessed.

### Practical Algorithm and Recommendations

**Fig. 3:**
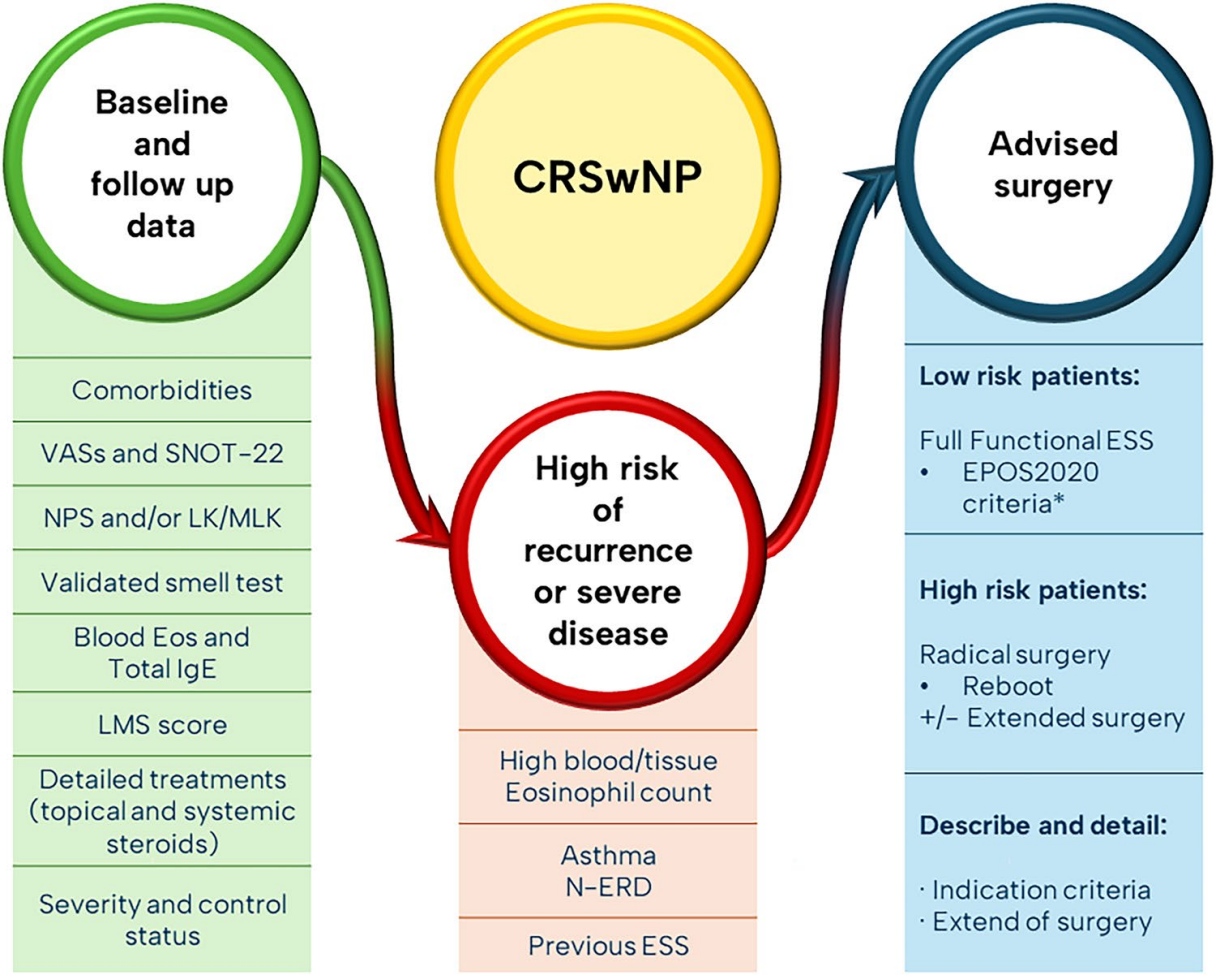
Practical algorithm and recommendations. VASs (Visual analog scales: obstruction, rhinorrhea, hyposmia, pressure/headache and global); NPS (Nasal Polyp Score); LK (Lund-Kennedy); MLK (Modified Lund-Kennedy); LMS (Lund Mackay System); N-ERD (Non-steroidal exacerbated respiratory disease); ESS (Endoscopic sinus surgery). *EPOS2020 criteria for functional surgery: Creates a sinus cavity that incorporates the natural ostium, allows adequate sinus ventilation, facilitates mucociliary clearance, facilitates instillation of topical therapies [1]

Based on the literature available, the findings of this review and considering the limitations of this study, Fig. 3 suggests a simple and practical algorithm. It aims to gather baseline and follow up data from CRSwNP patients, identify predictors of severe disease and recurrence, and suggest type of surgery along with data collection regarding indication and description of surgical treatment.

## Conclusions

This review of long-term outcomes following ESS in CRSwNP patients points out ESS as a safe and effective intervention, revealing long-term improvement in PROMs. Variables such as asthma, N-ERD, atopy, and blood eosinophils prove to be useful for tailored treatment and foresee severe and recalcitrant disease. Challenges in variables assessments highlight the need for standardization in questionaries (PROMs and QoL), NPS, loss of smell evaluation, precise definition of adequate treatment before surgery, ESS indication criteria, use of partly controlled and uncontrolled disease, surgery extent reporting system, revision surgery indication criteria and major variables valuable to evaluate the results of ESS in future research. Patient clustering offers insights into personalized approaches. Elevated IL-5 emerges as a potential predictor for revision surgery, suggesting a potential role for the indication of some monoclonal antibodies. While acknowledging limitations, this review establishes a foundational understanding of long-term outcomes, guiding future research and clinical decisions.

## Data Availability

No datasets were generated or analysed during the current study.
